# Supplementary material for: Rapid turnover of life-cycle-related genes in the brown algae
Source: Genome Biol. 2019 Feb 14;20:35. doi: 10.1186/s13059-019-1630-6 (PMC6374913; doi:10.1186/s13059-019-1630-6)
Supplement: Supplementary file 3 — Protein alignments, corresponding Gblocks html file and CDS sequences for the lineage conserved gametophyte-biased genes. (ZIP 2109 kb) [file 13059_2019_1630_MOESM3_ESM.zip › Con_GA_alignments/Ec-12_003300.aa_ali.fasta-gb.htm]

Ec-12\_003300.aa.fasta.trans.aa\_ali.fasta


## Gblocks 0.91b Results

Processed file: **Ec-12\_003300.aa.fasta.trans.aa\_ali.fasta**  
Number of sequences: **2**  
Alignment assumed to be: **Protein**  
New number of positions: **653** (selected positions are underlined in blue)

```
                         10        20        30        40        50        60
                 =========+=========+=========+=========+=========+=========+
Ec-12_003300     MTRLVGLDACPSVTSLDVSWNALTELDSACLEECKELWIVDASHNRLESLGGLSRVMALG
MSTRG.59311.1    ------------------------------------------------------------
                                                                             


                         70        80        90       100       110       120
                 =========+=========+=========+=========+=========+=========+
Ec-12_003300     YLNLSSNFIPLDGLRPLSRAHILELFLGSGRSSKERRRTLCVLPNLWVLDDEFVTAEERR
MSTRG.59311.1    ------------------------------------------------------------
                                                                             


                        130       140       150       160       170       180
                 =========+=========+=========+=========+=========+=========+
Ec-12_003300     IADDNHQHNGRGWGATSDHLLPTWNQSSPKRDKTRDKNSSTAIASPSSIVVVKDAGRHHP
MSTRG.59311.1    ------MSTGDTTGAVPDG-----------------------------IATSAPTAR-PP
                                                                             


                        190       200       210       220       230       240
                 =========+=========+=========+=========+=========+=========+
Ec-12_003300     PQKQKESACKSGFVDLETQGHLTQDFYENVVWKIPSRAVILFTRSPACPELVAWQTSPMD
MSTRG.59311.1    PGEEEAGVARSGLPGAQAR-PLGI-----GLTRLCRRAVILLTRSPTCPDLVAWQPGPME
                                                     ########################


                        250       260       270       280       290       300
                 =========+=========+=========+=========+=========+=========+
Ec-12_003300     QPAYDQMEPLLARAKLSHDDLHPTSVSFGGEVDGDHRRDGLGGGSVVPSRHRSPRTPCFH
MSTRG.59311.1    QRTYDQMKPLLAKAHMSHRDLYPQSE-FSHEFRGNSRAVSMTRGFSMASDLHSPSTPRMN
                 #########################  #################################


                        310       320       330       340       350       360
                 =========+=========+=========+=========+=========+=========+
Ec-12_003300     FDPLPADDDRG------DRGGVHATQLA--DSVDSLPLPDAGFWRASIRRPRSGERVQVL
MSTRG.59311.1    LDSSLSGSDIERLGPGEDRCSQQYARHEQPFSIEPLQPPDPGCWEANLRRPKAGERVQVL
                 #########                      #############################


                        370       380       390       400       410       420
                 =========+=========+=========+=========+=========+=========+
Ec-12_003300     CDTWSVIVTVDPEAFLVTVAPFEGIQTSAYRGEARIDSRDLYYDPRGWWSHVPAIAGNAT
MSTRG.59311.1    HDVWAAIIVVKPTTFSVVVAPFDGIQAPPYRGRPRIDSRDLYYDSRGWWRHIPALNNTTP
                 ######################################################      


                        430       440       450       460       470       480
                 =========+=========+=========+=========+=========+=========+
Ec-12_003300     AGPSRLHRQSRGLSASGLPRGAGSLNEALEEGKGDEPSQILNGRPSGGDREVVVDSSRNY
MSTRG.59311.1    VASGRLHRVNRGLSASGLPWGSGSLNEPLENPGVSDPRRSLSRCTSDVD---VV------
                     #############################################           


                        490       500       510       520       530       540
                 =========+=========+=========+=========+=========+=========+
Ec-12_003300     TDLLLPSAAQQLSQPASSAAAPVSKSLSPAVSTEPSTARGLTASSNAAAAPSNPSPAPSV
MSTRG.59311.1    ---DRPETDQQISPESTGAVTGGRRALTAALSPSSTTRNLAAPRAHTVAPPNSLSLAPTV
                      ################################           ############


                        550       560       570       580       590       600
                 =========+=========+=========+=========+=========+=========+
Ec-12_003300     LRSHRCPNNNVVDVFSAEGSWDPVFVVAPPRLVGLQNQTAARRMGGRRARQVAWYRIDGP
MSTRG.59311.1    L-RHRCLSDDV-GVFSAEGSWDPVFVVAPARLVGLQNQTAVRQMGPRRALQVAWRPIEGP
                 #            ###############################################


                        610       620       630       640       650       660
                 =========+=========+=========+=========+=========+=========+
Ec-12_003300     EVSVAPRPTMPRCSSLRMPFRVGDTPSRHLQQSASTSSVPRLSSKQAPLRLAAVAPSHGG
MSTRG.59311.1    EGSVAPRPTMPRCSSLTTSAGAGAIRCPILQQSASTPSIPGIFDKRPPTRWASVAPSHE-
                 ##########################################################  


                        670       680       690       700       710       720
                 =========+=========+=========+=========+=========+=========+
Ec-12_003300     GNSVYARFSQSRGLSVPSINTNSCSMPAAKKLPTAADEAWLELQREMGSLKADNRMLIRD
MSTRG.59311.1    SSSVNKRFPLSAALSASSINTSSCSAVAAGVPVPPTDEAWLDLQKEMGSLKADNRMLIRG
                   ##########################################################


                        730       740       750       760       770       780
                 =========+=========+=========+=========+=========+=========+
Ec-12_003300     SSQGGGGGGGECINTSTISAS-SPAMSVVTKSLAENSTVGAQATTDMLAKTLTRTLDSPL
MSTRG.59311.1    GSQSGSTDTGLVSKAQALLRTSGSAMSVMTRSFAENSTVCAEATADLLAKTLSLGLDDPV
                 ##########              ####################################


                        790       800       810       820       830       840
                 =========+=========+=========+=========+=========+=========+
Ec-12_003300     SAWKGGNTVSATATVFELTQVGGRPEKDAG--ECASSPTSVSLTHETTRSTDNWRVDNQQ
MSTRG.59311.1    SAWKGGRQENTPTTVFELTQVDGKPKTSTEGVTFATCSTPVRDREATPTRGEASEIVSQK
                 #########################         #############           ##


                        850       860       870       880       890       900
                 =========+=========+=========+=========+=========+=========+
Ec-12_003300     QEVSPPEECSKQVRVAEGD--KPDTAASSHRRRRIPDDGNSTAASATTRKWKPNAFRPLE
MSTRG.59311.1    CAVSMSQESLARAAAGEDQRALPQRNQSARCRRRHSQEGISTTATTPSTTWKPNVHRPLD
                 #################     ######################################


                        910       920       930       940       950       960
                 =========+=========+=========+=========+=========+=========+
Ec-12_003300     WFPVPGRQVFRVDPNDVNAAPAAEPVGRPVESGTSNTPNVPPEGQGAEGGGGGGGGGDGG
MSTRG.59311.1    WFPVPGRQVLLVDPNDMKAAATAAAAKATADREAAIN---TSEGDDFRTTGESASGDECS
                 #######################                   ##################


                        970       980       990      1000      1010      1020
                 =========+=========+=========+=========+=========+=========+
Ec-12_003300     GGGGNCGSSERQAVGGEDTVEQDKAGRRGGQAKEKIRIESGKGQPGNGS---PSTRSPFP
MSTRG.59311.1    QDRGAGRAPAQKLVRGGGEIGKRPEKTKSRQAEGRGGFGRDMGESRDRSEILLSARSGFP
                 ####                                                 #######


                       1030      1040      1050      1060      1070      1080
                 =========+=========+=========+=========+=========+=========+
Ec-12_003300     TAAVLNNGLLSPATNRDMGVRPTIWTRSRRARSSRSLHSAGAFRALDRQKPNGLLADALR
MSTRG.59311.1    SAAVLADISLSPKNDKS-------------------------------------------
                 ############                                                


                       1090      1100      1110      1120      1130      1140
                 =========+=========+=========+=========+=========+=========+
Ec-12_003300     PKSPDSLGVITSFSTQVRLEIDPHDVRSRGFHRREEEHCRETRQCVEASDVSSGLAVTPG
MSTRG.59311.1    -----------------------AEARARG---------------------PSGQCKAPG
                                                                     ########


                       1150      1160      1170      1180      1190      1200
                 =========+=========+=========+=========+=========+=========+
Ec-12_003300     VRPMVSGPTGQRPARTKAVVPHWQHDKRHPARRQPQCQRLTISGWEGAEGAGSTSGDLMR
MSTRG.59311.1    CHAPASPANGVPPRKSSGALDLTSHS---------------VHGWP--------------
                 #############                                               


                       1210      1220      1230      1240      1250      1260
                 =========+=========+=========+=========+=========+=========+
Ec-12_003300     LPTFKPSESPELESSCRNLGAVSDKGGPGAPPSGGILKSSLARGKRGGARVKARPASPHH
MSTRG.59311.1    ----------------------ESKPGQG-------------------------------
                                                                             


                       1270      1280      1290
                 =========+=========+=========+=
Ec-12_003300     SDSGLDECFSISSAATSNNNSCCGWRKSDSM
MSTRG.59311.1    ----------------------------AQX
```

```
Parameters used
Minimum Number Of Sequences For A Conserved Position: 2
Minimum Number Of Sequences For A Flanking Position: 2
Maximum Number Of Contiguous Nonconserved Positions: 8
Minimum Length Of A Block: 10
Allowed Gap Positions: None
Use Similarity Matrices: Yes
```

```
Flank positions of the 15 selected block(s)
Flanks: [217  265]  [268  309]  [332  414]  [425  469]  [486  517]  [529  541]  [554  658]  [663  730]  [745  805]  [815  827]  [839  857]  [863  923]  [943  964]  [1014  1032]  [1133  1153]  

New number of positions in Ec-12_003300.aa.fasta.trans.aa_ali.fasta-gb:  653  (50% of the original 1291 positions)
```
